# Evaluation of a collaborative model for successful implementation of a National CD4 enumeration EQA program in Cameroon

**DOI:** 10.1038/s41598-021-91015-7

**Published:** 2021-06-02

**Authors:** Bertrand Sagnia, Sandra Kiazyk, Adrienne F. A. Meyers, Margot Plews, Tamsir O. Diallo, Samuel Martin Sosso, Georgia Ambada, Rachel Kamgaing, Nadesh Nji, Paul Sandstrom, Blake T. Ball, Godwin Nchinda, Alexis Ndjolo

**Affiliations:** 1 Chantal BIYA International Reference Centre for Research on Prevention and Management of HIV/AIDS (CIRCB), Yaounde, Cameroon; 2 National HIV and Retrovirology Laboratories, JC Wilt Infectious Diseases Research Centre, Public Health Agency of Canada, Winnipeg, MB Canada; 3 Department of Medical Microbiology and Infectious Disease, University of Manitoba, Winnipeg, Canada

**Keywords:** Immunology, Microbiology, Health care, Medical research

## Abstract

Participation in an EQA program is critical to the quality assurance process. Reliable and precise CD4 T-cells enumeration are essential to improve the clinical management of patients by evaluating the disease progression and by monitoring the effectiveness of ART in HIV-patients. The CIRCB, CD4 reference laboratory, in collaboration with the Canadian QASI-program, recruited sites, distributed and analyzed CD4-panels in 61 sites across Cameroon. A trend and performance analysis in the pre-analytical, analytical and post-analytical phases was performed. Continuous training and corrective actions carried out from 2014 to 2018 increased the number of participating sites from 15 to 61 sites, the number of unacceptable results decreased from 50 to 10%. Specific challenges included errors in pre analytic (17.5%), analytic (77.0%) and post-analytic (5.5%) phases. This EQA requires the application of good laboratory practices, fluidic communication between all the stakeholders, continuous training, application of specific on-site corrective measures, and timely equipment maintenance in order to avoid repetitive errors and to increase laboratory performance. It could be extended to other HIV-1 testing like viral load and EID point-of-care. Partnership with QASI serve as a model for implementation of a successful EQA model for resource limited countries wanting to implement EQA for HIV testing and monitoring in alignment with 90–90–90 targets.

## Introduction

Globally, more than 38.0 million people are infected with Human Immunodeficiency Virus (HIV) and approximately 540,000 people in Cameroon are living with this infection at a prevalence of 3.6% in 2018^1^. In 2014, UNAIDS established the 90:90:90 goals for HIV testing, accessible therapy, and viral suppression by 2020^2^. These targets were followed by new guidelines from the WHO in 2016, which recommend VL as the preferred approach to ART monitoring compared to clinical or immunological criteria^3^. Where previously HIV monitoring has relied heavily on CD4 enumeration, these new global targets for HIV elimination rely heavily on viral load monitoring^4^. Implementation of viral load monitoring programs however is still hindered by several barriers and delays, and for many rural zones in resource-limited countries where VL monitoring is unavailable, CD4 monitoring remains the primary mechanism for patient monitoring and clinical management^5^. In developing and in low income countries, CD4 count still used in the management of HIV infected patients^6,7^. CD4 + T-cell lymphocytes are a primary target for HIV infection and these cells are depleted throughout the course of the disease. CD4 T cell counts are therefore an important indicator of a patient’s immune and clinical status. In addition, CD4 testing is critical at the time of initial time of diagnosis in order to evaluate risk and guide the treatment of opportunistic infections^4^.

Traditionally, CD4 T cell enumeration has been performed using conventional flow cytometry instruments^8,9^. Implementation of point-of-care CD4 testing instruments has shifted the conventional delivery of testing, and has allowed for the provision of testing services to those in remote, marginalized, and resource-limited settings^10–14^. Regardless of the technology used, the success of any diagnostic or clinical monitoring program, including CD4 enumeration, is the availability and accuracy of testing^15,16^. In resource-limited countries, where a majority of laboratories are not accredited, participation in an external quality assurance (EQA) program mainly limited to reference laboratories, is critical and contributes to ensuring delivery of accurate and reliable diagnostic test results^15,17,18^.

Various EQA programs for CD4 enumeration exist internationally and include QASI-LI in Canada, UKNEQAS in England, and AFREQAS in South Africa^19–22^, among others, both commercial and non-commercial. Many of these EQA programs are cost prohibitive to resource-limited countries already committing their resources to procuring the testing kits. Independent provision of EQA services offers a solution to this financial constraint, however there are still many major challenges for independent EQA implementation including: the lack of knowledge about quality management practices, procurement of EQA materials, lack of skills for EQA management, inadequate and unsustainable funding, and transportation challenges^23,24^. As a solution to this, The QASI-LI program, through the Public Health Agency of Canada offers EQA for CD4 enumeration at no cost, with a goal of providing training and support towards the goal of becoming independent. Together with QASI, the Chantal BIYA International Reference Center for Research on Prevention and Management of HIV AIDS (CIRCB) initiated a collaborative working relationship to develop a strong CD4 quality assessment program within the CIRCB to oversee CD4 testing throughout Cameroon.

Here we present an evaluation of our program, from 2014 to 2018, providing evidence that EQA programming in Cameroon is leading to the delivery of more accurate results from the participating sites and describe the successes and challenges of quality management implementation in HIV/AIDS CD4 testing laboratories in Cameroon. This will also serve as the basis for launching additional testing options such as POC viral load (VL) and early infant diagnosis (EID) initiatives and to have them linked to the same stream of quality assurance services in collaboration with QASI and can be used as a model for other countries to implement their own national / regional EQA programs. There is no similar program in all the central Africa sub region.

## Materials and methods

### QASI

QASI, the Public Health Agency of Canada’s International Program for Quality Assessment and Standardization of Indicators relevant to HIV/AIDS is hosted at the National HIV and Retrovirology Laboratories in Winnipeg, Canada. QASI provides EQA through administration of proficiency testing (PT) panels and comprehensive corrective and preventative action as required in English and French. QASI strives to ensure accurate and reliable CD4 enumeration through the provision of proficiency testing panels, external quality oversight and comprehensive and preventative action recommendations. QASI-LI administration works closely together with a country coordinator and assist in developing and launching in country a National EQA program^15^.

### Study sites

**Figure 1:**
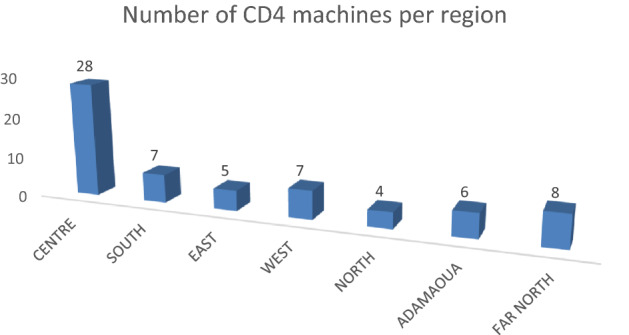
Distribution of participants in the CD4 EQA programming within Cameroon.

The CIRCB, as a reference CD4 laboratory, was mandated by the Ministry of Public Health to implement a national CD4 EQA program in Cameroon. This Laboratory act as the QASI national coordinating centre for CD4 T cell enumeration within Cameroon and is responsible for receiving, distributing and analyzing samples sent from QASI-LI headquarters in Canada to lab sites across Cameroon. Over 4 years, 2592 EQA samples were sent to CD4 sites present in seven regions of Cameroon (Fig. 1). Each site received EQA panels (two samples) three times per year. In Cameroon, instruments for CD4 enumeration are limited and spread around the country. Instruments include conventional flow cytometers and point-of-care (POC) testing platforms like PIMA.

### Panel distribution and testing

The proficiency testing (PT) material is a commercially produced stable whole blood product. QASI-LI orders this material in prefilled 1-ml vials, labeled with unique QASI-LI specimen identifiers. These samples are shipped three times each year from QASI-LI headquarters in Winnipeg, Manitoba, Canada. Each participating laboratory site receives two distinct samples for testing; they include one with a mid CD4 count level and one with a low CD4 count level^25^. The material is shipped in a Styrofoam box including ice packs, as the samples are maintained close to 4 °C during transportation to preserve sample integrity. Samples are packaged in a sealed plastic bag, with suitable absorbent material to absorb liquid in case of breakage. Upon receipt of samples, laboratories are instructed to acquire and analyze as soon as possible using their standard operating procedure for testing human patient samples.

### Data analysis and reporting

Data on the percentage and absolute count for CD3, CD8 and CD4 + T-cells is entered and submitted to the QASI-LI Website, LymphoSite (https://qasi-lymphosite.ca). To protect confidentiality, a unique user name and password is assigned to each laboratory. The online data submission process collects information about dates of specimen arrival, date of analysis, specimen processing details, and participant results. All submitted data are tabulated to obtain the group mean value and standard deviation (SD) value for each marker. Results exceeding the aggregate mean by greater than two standard deviations are considered outliers. These outliers are excluded from the analysis. Aggregate group mean and standard deviation are then recalculated and used to assess individual laboratory performance against this calculated mean. Individual laboratory performance is evaluated by the Standard Deviation Index (SDI). The SDI is calculated by dividing the residual value by the aggregate SD. SDI indicates how well the reported value compared with the aggregate group mean for a given cell subset value. A positive or negative sign indicates that the reported value is higher or lower than the aggregate mean value obtained by the group respectively. The laboratory’s performance is satisfactory when the results fall within ± 2 S.D.I. A performance report is provided for each individual laboratory. The regional or country coordinator will receive a detailed report for their country which includes an overall summary of the session for the Coordinator and more detail with respect to what is recommended for corrective actions for sites which do not pass.

### Corrective action

Corrective action is a critical and mandatory requirement of the QASI-LI program to help participating sites resolve challenging situations. The sooner corrective action measures are implemented, the shorter the time the site will be reporting inaccurate clinical results and impacting patient care and management. The QASI program collects data on various errors including errors in different phases: pre-analytical phase, analytical phase and post analytical phase. Errors recorded include those due to delayed sample transportation and preservation, expired reagents, pipetting and pipette calibration, incorrect sample identification, request procedure error, sample mix-ups. Post analytical and analytical phase errors, such as methodology used/reported, result of errors with sample handling/processing, error codes from instruments received during testing and lack of appropriate data reporting are also recorded. When required, corrective actions were applied to testing sites with unsatisfactory performance. If needed, onsite visits are made by the country coordinator to identify sources of error. During these visits, each step of laboratory processing is observed and reviewed, including standard operating procedures, pipetting techniques and pipette calibration protocols, sample handling, instrument handling and maintenance and technician competency.

### Longitudinal data analysis of EQA performance

An analysis of this program was performed to assess the growth and success of the program. Data over the course of 11 EQA sessions (QASI sessions 48 to 58) from 2014 to 2018, data was extracted to analyze trends in participation, performance, error sources, and charted for visualization. All data was extracted from the QASI-LI website database and analyzed using Microsoft Excel.

## Results

In Cameroon, 10 regions have health facilities to perform CD4 enumeration for HIV infected patients. EQA of CD4 enumeration for these health facilities is managed by two programs; Cameroon CDC in the North-West, South-West and Littoral regions supported by the Centre for Disease Control (not discussed in this paper) and Cameroon National EQA Program in the Far-North, North, Adamaoua, West, Centre, South and East regions support by CIRCB.

**Figure 2:**
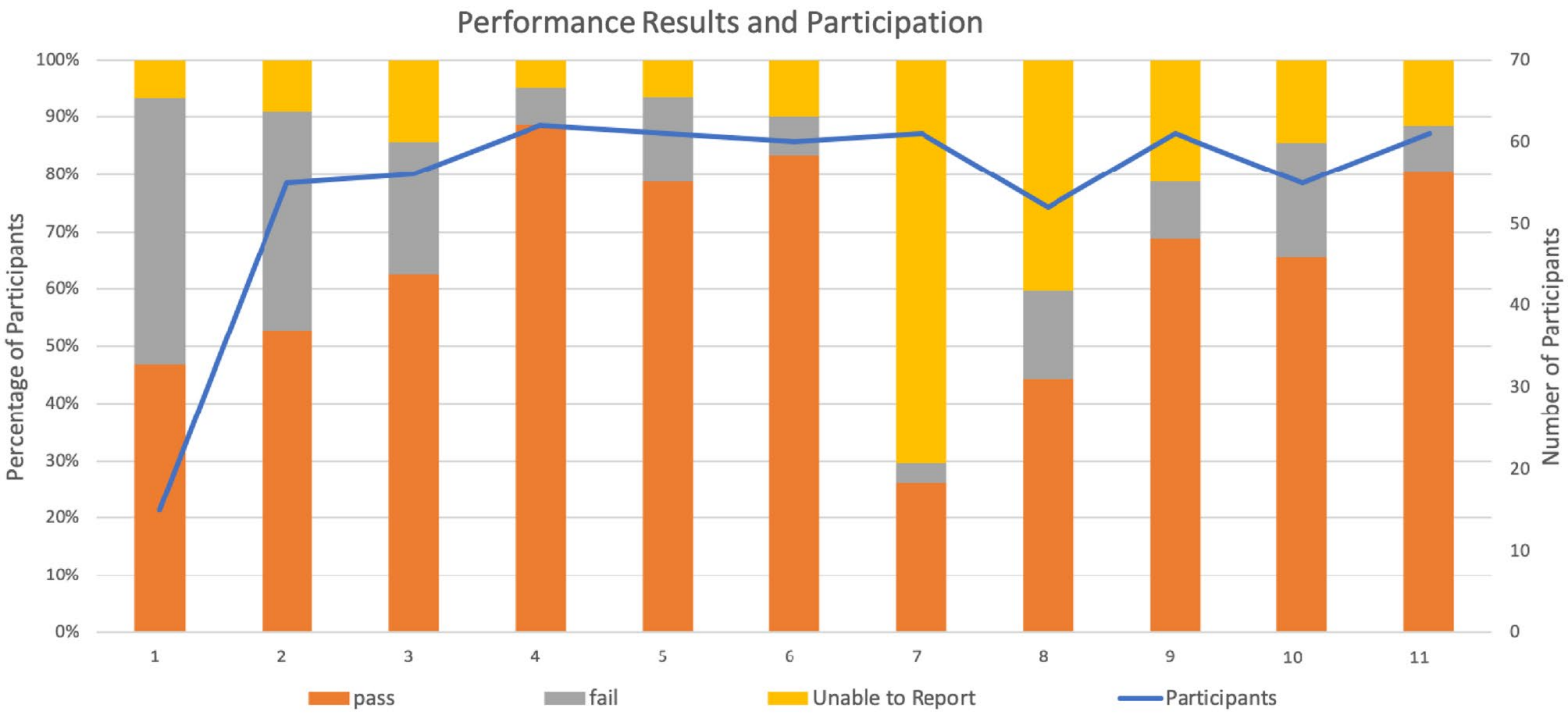
Performance of all participants. Indicated are pass, fail, and unable to report percentages for 11 EAQ sessions. The blue line indicates the number of participants per session. Unable to report means that these labs registered for a session but did not send results due to no login/broken instrument/no reagents/expired reagents, therefore these labs did not run the sample.

Figure 1 shows the distribution of the 65 regional sites participating in the National EQA program coordinated by the CIRCB in collaboration with QASI-LI across Cameroon. These sites are located in both urban and rural areas, but as seen a majority of participants are centrally located. A variety of CD4 enumeration platforms (Table 1), both conventional and POC, including PIMA from Alere (now Abbot), FACSCount from BD Biosciences and CyFlow from Partec (now Sysmex-Partec) are registered in this program. The participation rate in the National EQA program within Cameroon started small (shown in Fig. 2) but rapidly increased from the pilot session (QASI-LI session 38) in 2014, with 15 participating sites to 65 participating sites at present.

**Table 1:** Data on pass and failure rates for all instruments further broken down by instrument type over the 11 evaluated EQA sessions.

	All instruments			FACSCount			CyFlow			PIMA			Others		
	N°	Pass	Fail	N	Pass	Fail	N°	Pass	Fail	N°	Pass	Fail	N°	Pass	Fail
1	**14 (100)**	*7 (50)*	*7 (50)*	**8 (57)**	*3 (37.5)*	*5 (62.5)*	**3 (21)**	*2 (67)*	*1 (33)*	**2 (14)**	*1 (50)*	*1 (50)*	**1 (7)**	*1 (100)*	*0(0)*
2	**50 (100)**	*29 (58)*	*21 (42)*	**16 (32)**	*9 (56)*	*7 (44)*	**13 (26)**	*5 (38)*	*8 (62)*	**17 (34)**	*11 (65)*	*6 (35)*	**4 (8)**	*4 (100)*	*0 (0)*
3	**48 (100)**	*35 (73)*	*13 (27)*	**16 (33)**	*11 (69)*	*5 (31)*	**11 (23)**	*6 (55)*	*5 (45)*	**19 (40)**	*16 (84)*	*3 (16)*	**2 (4)**	*2 (100)*	*0 (0)*
4	**59 (100)**	*55 (92)*	*4 (7)*	**11 (19)**	*10 (91)*	*1 (9)*	**14 (24)**	*12 (86)*	*2 (14)*	**32 (54)**	*31 (97)*	*1 (3)*	**2 (3)**	*2 (100)*	*0 (0)*
5	**57 (100)**	*48 (84)*	*9 (16)*	**9 (16)**	*8 (89)*	*1 (11)*	**13 (23)**	*10 (77)*	*3 (23)*	**33 (58)**	*28 (85)*	*5 (15)*	**2 (4)**	*2 (100)*	*0 (0)*
6	**54 (100)**	*50 (93)*	*4 (7)*	**11 (20)**	*11 (100)*	*0 (0)*	**8 (15)**	*7 (87)*	*1 (13*	**33 (61)**	*30 (91)*	*3 (9)*	**2 (4)**	*2 (100)*	*0 (0)*
7	**18 (100)**	*16 (89)*	*2 (11)*	**6 (33)**	*6 (100)*	*0 (0)*	**2 (11)**	*2 (100)*	*0 (0)*	**9 (50)**	*8 (89)*	*1 (11)*	**1 (5)**	*0 (0)*	*1 (100)*
8	**31 (100)**	*23 (74)*	*8 (26)*	**9 (29)**	*5 (55.5)*	*4 (44.5)*	**5 (15)**	*4 (80)*	*1 (20)*	**15 (48)**	*13 (87)*	*2 (13)*	**2 (6)**	*1 (50)*	*1 (50)*
9	**48 (100)**	*42 (87)*	*6 (13)*	**5 (10)**	*5 (100)*	*0 (0)*	**9 (19)**	*7 (78)*	*2 (22)*	**32 (67)**	*29 (90)*	*3 (10)*	**2 (4)**	*2 (100)*	*0 (0)*
10	**47 (100)**	*36 (76)*	*11 (24)*	**6 (13)**	*4 (67)*	*2 (33)*	**7 (15)**	*2 (28)*	*5 (72)*	**32 (68)**	*29 (90)*	*3 (10)*	**2 (4)**	*1 (50)*	*1 (50)*
11	**54 (100)**	*49 (90)*	*5 (10)*	**6 (11)**	*6 (100)*	*0 (0)*	**8 (15)**	*5 (62)*	*3 (38)*	**37 (68)**	*36 (97)*	*1 (3)*	**3 (6)**	*2 (66)*	*1 (34)*

Numbers in bold indicate absolute instrument numbers included and percentages of total in parentheses below. Numbers in italics indicate pass and fail numbers and percentages of that instrument group in parentheses.

Figure 2 also displays overall performance rate (number and percentage) of participating sites who Passed, Failed or were Unable to Report results for each QASI-LI session. Although the pass rate among those able to report for each session ranged from 50 to 95%, we observed an overall rapid increase in the number of participating sites who reported correct results across all sessions following implementation of the program.

**Figure 3:**
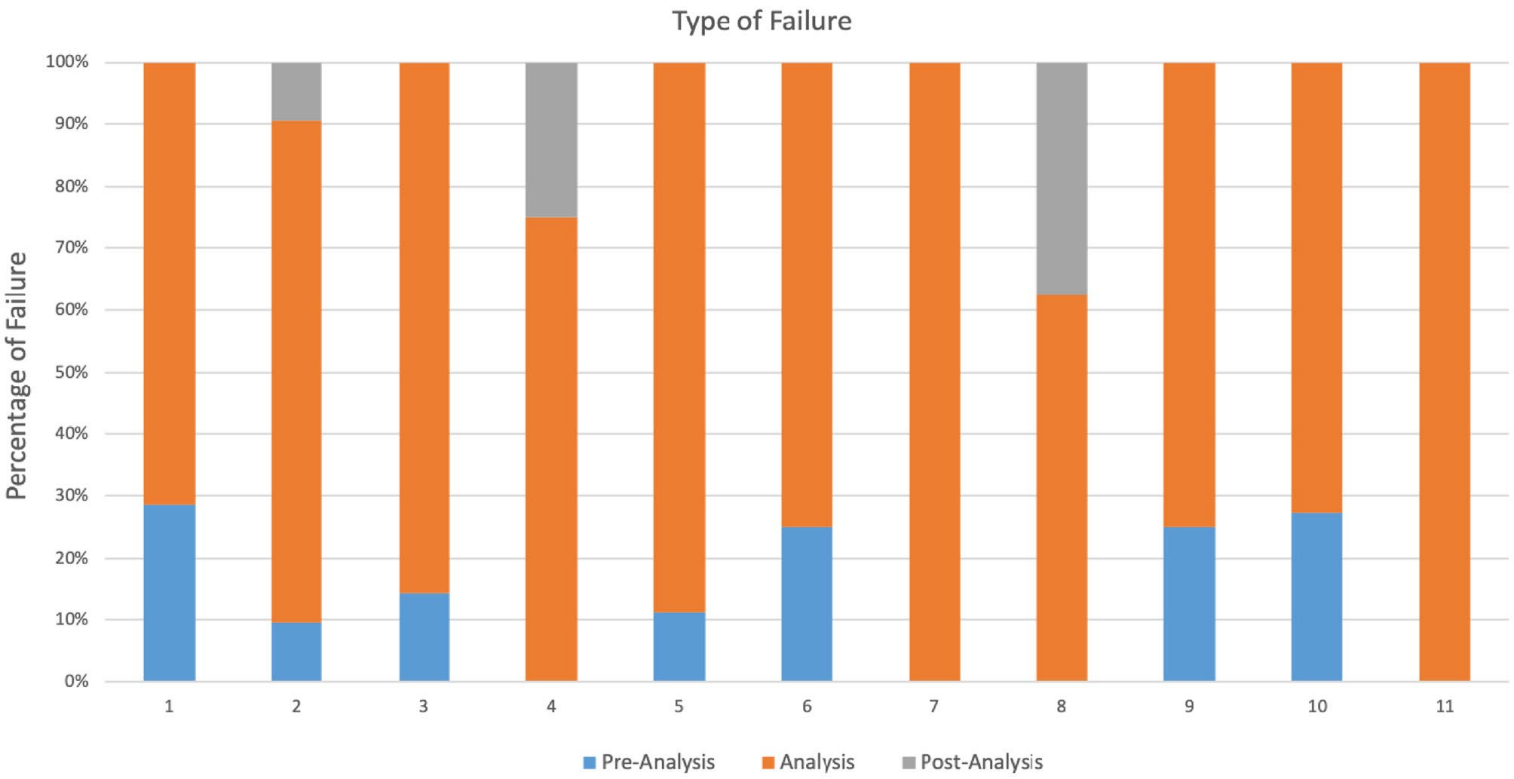
Phase of failures present across sessions. The data here do not include those who were unable to report. This figure is only based on labs who submitted a result.

For participating sites who fail a session because their reported results are outside of the acceptable range, a major part of the EQA program is to assess the reason for incorrect results in order to implement successful corrective and remedial measures. Errors were identified in the pre-analysis, during analysis, and post-analysis phases. Figure 3 represents the distribution of these errors across the 11 sessions among participating sites who reported results outside of the acceptable range. The majority of failures were in the analysis phase, which ranged from 63.5% to 100% in any given session as seen in Fig. 3. Failures during the pre-analytical phase varied from 0 to 28.5% while failures during the post-analysis phase varied from 10% to 37.5% in a session.

**Figure 4:**
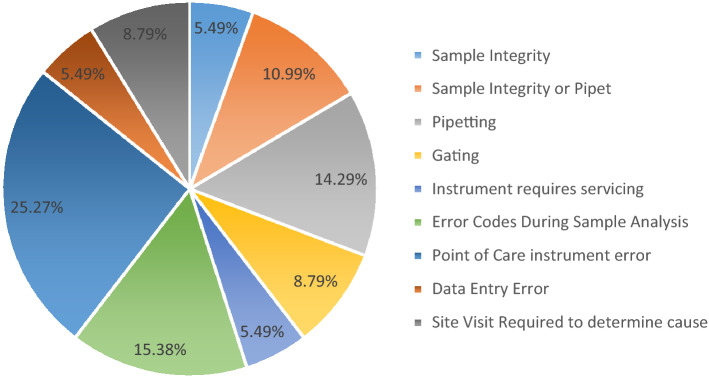
Observed types of errors across all 11 evaluated EQA sessions: these errors were observed when participants returned an incorrect CD4 result whereas errors made by participants who have not returned a CD4 result (unable to report) are excluded.

Figure 4 further summarizes the distribution of errors seen across all 11 sessions. In order to identify if failures were higher in labs using a particular instrument or a POC device, we calculated pass and fail rates across sessions according to the CD4 enumeration platform used.

Table 1 identifies the variety and number of instruments (both conventional and POC) used in this program with pass and fail rates observed for those instruments over the evaluated sessions.

**Figure 5:**
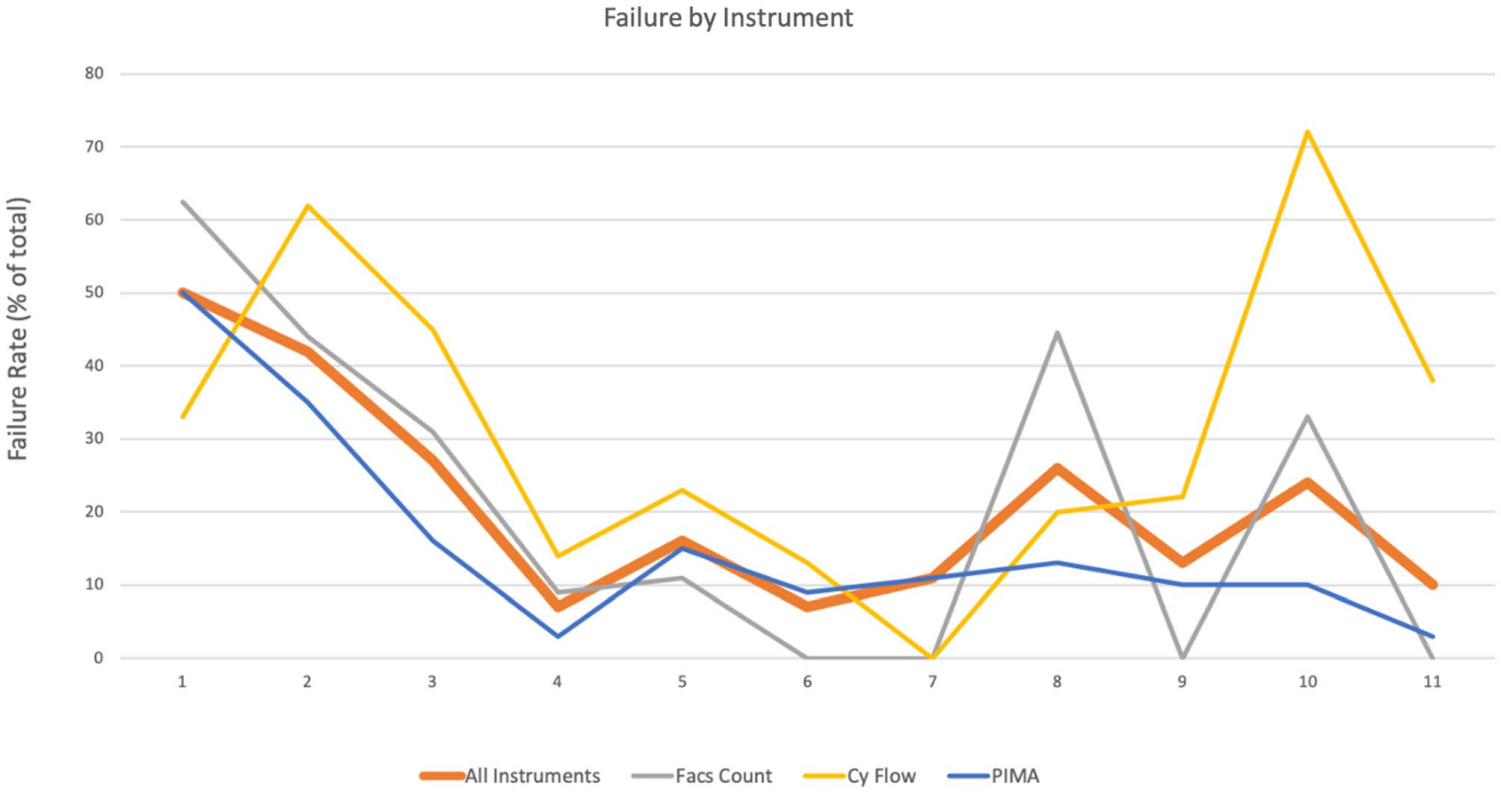
Failure Rates for 11 evaluated EAQ sessions broken down by type of instrument used. Failure rates as a percentage of the total are shown for all combined instruments, FACSCount instruments, CyFlow instruments and PIMA instruments. Data shown includes only participants who returned a result, not those who were unable to report a result.

Overall, the number of conventional flow cytometry instruments, including the FacsCount and CyFlow, reduced in number across sessions, while the number of sites utilizing the PIMA analyzer increased from 2 sites to 37 over the evaluated timeframe. (As some sites do not always use the same instrument to participate in the EQA program, we do not always have consistent numbers and instruments used across sessions). Figure 5 shows the failure rates for each of the different CD4 enumeration instruments used.

The orange line representing all instruments combined serves as the baseline and is used as a reference for all comparisons. CyFlow and FACSCount users often had failure rates higher than the overall instrument failure rate. In contrast, failure rates observed for PIMA users were generally lower.

## Discussion

Participating health facilities in this National EQA program include those from both the public and private sector located in urban and rural areas. Also the diversity of machines results in a diversity of manufactures/suppliers, for supply of both reagents and instrument maintenance as well as requiring operators to display competence on multiple platforms. The quick growth in participation observed was due in large part to increased communication and expanded engagement efforts of the country coordinator with the participating labs. It’s observed also the fact that, the number of testing sites within a region is not proportional to the number of HIV patients accessing service. An excellent example of this is in the case of HIV positivity in PMTCT site laboratories. As seen in Fig. 1, in the North region there are only four CD4 enumeration instruments used to test more than 700 expectant HIV positive pregnant women while in the West region there are seven CD4 instruments for less than 700 expectant HIV positive pregnant women in 2015^26^. This clearly illustrates that EQA coverage across the country is not proportional to the amount of testing at each site. A goal will be in the future to increase the participant’s number in the others regions (West, South and North).

The corresponding failure rates observed decreased with time, particularly in the 4^th^ session following the signing of instrument maintenance contracts by the Ministry of Public health. In addition, failures decreased over time due to response to corrective/remedial action recommendations resulting in increased training and on-site visits from the coordinating Centre. These trainings and on-site visits were extremely important as they allowed for careful review and consideration of conditions and practices in place at each site.

In sessions, 7 and 8 there was a large proportion of participating sites that were Unable to Report results as seen in Fig. 2. Sites with this status are those with broken instruments, expired reagents, no reagents or were unable to report for other reasons. The high rates of participating sites that were Unable to Report results during these sessions reflect the reality of CD4 testing in resource-limited settings, where funding for procurement of reagents is not always stable.

The high percentage of errors observed in analytical phase commonly occur during sample preparation and include pipetting errors, or they occur during sample acquisition/analysis and inaccurate gating technique or instrument errors. For some instruments, such as the Cyflow, the pipet calibration and pipetting technique play an important role to have accurate results. The Cyflow instrument is a volumetric instrument, it is recommended to have a good precision when pipetting blood. It’s important for these labs frequently as they can to verify their pipet calibration and pipetting technique by assessing precision and calibrating pipettors. In addition, an important aspect of training included learning to override the automation and apply manual techniques to achieve the best testing possible. This is in contrast with the routine clinical analysis where most of the errors occurred in the pre-analytical phase which involving too many professionals, such as physicians, specialists of laboratory medicine, nurses, laboratory technicians and phlebotomists and including patient preparation and identification error, inappropriate specimen transportation, inadequate and inappropriate specimen collection tubes and storage, inappropriateness of test order, timing errors in sampling and preparation, hemolytic and lipemic blood samples. In our context of EQA program, samples received are already identified, ready to be stained for analysis^27–29^. Failures during the pre-analytical phase varied from 0% to 28.5% in a session and were mainly the result of issues related to sample integrity during transport and or preservation. This is a challenge as samples arrive from Canada and must be kept cold and redistributed across the country. Failures during the post-analysis phase varied from 10% to 37.5% in a given session and were mainly the result of errors during data entry either onto the paper-copy result submission form or onto the electronic website for result collection. Post-analysis errors also made a small proportion of total non-conformances and consisted of transcription errors which can be resolved by implementing verification steps into the data submission process.

An important component of the QASI EQA scheme is to perform remedial action to identify sources of error in order to implement corrective measures in order to attain successful results. Having knowledge of all of the different instruments being utilized across the country we wondered if certain instruments had a disproportionate amount of false results associated with their use. As shown in Fig. 5, the PIMA point-of-care instrument had consistently overall lower failure rates when compared to traditional cytometry instruments like the CyFlow or smaller cytometers like the FACSCount. The more erratic failure rates for FACSCcount and Cyflow may also be the consequence of their lower numeric representation in the survey as compared to PIMA as observed in Table 1. Importantly, uptake of these POC instruments has been increasing across Africa, and observed in our program as well. Starting with only 2 PIMA instruments in the program in session 1 and reaching 37 instruments in session 11 representing 70% of the instruments in the program. Given their ease of use and a consistently low failure rate in our program, these POC devices should be used for any expansion/new testing sites.

We have learned through our experience that ongoing communication with all participant laboratories is essential. While the initial training at the reference laboratory is a good start, it alone is not sufficient, and follow-up visits are essential. Every participating site has their own unique challenges that require consistent communication to both anticipate and overcome. The role of the country coordinator is to provide adequate and ongoing information to its participants while evolving in an effort to meet participant satisfaction and changing needs.

This study provides evidence that the implementation of the QASI CD4 EQA program within the CIRCB has helped to improve the reliability of CD4 + T-lymphocyte determinations made across Cameroon. This helps to ensure accurate delivery of results, which is of high clinical importance for patient care and management^30^. This type of oversight is becoming increasingly important as Cameroon scales up its national ART access program for people living with HIV/AIDS. This national EQA program has thrived despite numerous challenges, requiring years to reach its present status. It should be considered a significant advancement for Cameroon considering most of the laboratories that are part of the Cameroon National EQA were previously not participating in any other certification program. We would like to expand similar programming to other countries around the Central Africa Sub region in a sustainable manner, thus assisting neighboring nations to implement the improvements in quality of testing that we have observed. In addition, this EQA program on CD4 enumeration can be used as a model for other HIV testing and monitoring tests.

Ultimately, the CIRCB plans to enroll sites performing POC testing for VL and EID into the QASI-VL and EID programs for evaluation further strengthening the goals of CIRCB to provide comprehensive EQA programmatic needs to all HIV testing/monitoring sites in Cameroon. To reach the UNAIDS target of 90–90–90 and for bringing the AIDS epidemic to an end, especially in low and middle income countries, it is critical to improve the quality management systems, strengthen laboratory management, provide affordable external quality assurance and accreditation schemes, and build local capacity.

This is the first evaluation of an EQA program implementation in Cameroon and the first time that Cameroon has introduced the system of panel distribution and external quality control evaluation of performance for CD4 via an EQA Coordinating Center with the aim to build capacity in country like other functional EQA program in Africa^22^.

## Abbreviations

AFREQAS: African Regional External Quality Assessment Scheme
ART: Anti-retroviral therapy
CIRCB: Centre International de Référence Chantal BIYA pour la recherché sur la prise en charge du VIH/SIDA
EID: Early infant diagnosis
EQA: External quality assessment
HIV: Human Immunodeficiency Virus
POC: Point of care
QASI: Quality Assessment and Standardization of Indicators relevant to HIV/AIDS
UKNEQAS: United Kingdom National External Quality Assessment Scheme
UNAIDS: The Joint United Nations Programme on HIV/AIDS
VL: Viral load
